# Successful treatment of invasive tracheobronchial pulmonary aspergillosis with venovenous extracorporeal membrane oxygenation and combined systemic, intratracheal instillation of liposomal amphotericin B: a case report

**DOI:** 10.1186/s13256-022-03692-1

**Published:** 2022-12-20

**Authors:** Shuku Sato, Wataru Kamata, Kiyomitsu Fukaguchi, Shun Tsunoda, Tadashi Kamio, Hiroshi Koyama, Hideyasu Sugimoto, Yotaro Tamai

**Affiliations:** 1 Division of Hematology, Shonan Kamakura General Hospital, 1370-1 Okamoto, Kamakura, Kanagawa 247-8533 Japan; 2 Division of Critical Care Medicine, Shonan Kamaura General Hospital, Kamakura, Japan; 3 Division of Respiratory Medicine, Shonan Kamakura General Hospital, Kamakura, Japan

**Keywords:** Invasive tracheobronchial-pulmonary aspergillosis, Extracorporeal membrane oxygenation, Liposomal amphotericin B

## Abstract

**Background:**

Invasive pulmonary *Aspergillus* and invasive bronchial aspergillosis is a life-threatening opportunistic fungal infection that predominantly affects immunocompromised hosts. A case series and review found that the mortality rate of invasive bronchial aspergillosis is high, at about 40%, and 23.7% of invasive bronchial aspergillosis patients require mechanical ventilator management. There are few reports of life-saving cases with venovenous extracorporeal membrane oxygenation as rescue therapy in invasive pulmonary *Aspergillus* and invasive bronchial aspergillosis. Here, we report a case of invasive bronchial aspergillosis and invasive pulmonary *Aspergillus* that was successfully treated with venovenous extracorporeal membrane oxygenation, and combined systemic and intratracheal instillation of liposomal amphotericin B.

**Case presentation:**

We present the case of a 61-year-old Japanese man with invasive tracheobronchial-pulmonary aspergillosis while receiving chemotherapy for malignant lymphoma. Bronchoscopy revealed trachea covered with pseudomembranous necrotizing tissue, the culture revealed *Aspergillus fumigatus*, and the histological findings of pseudomembranous revealed fungal hyphae. The patient required venovenous extracorporeal membrane oxygenation because of respiratory failure for atelectasis and obstructive pneumoniae. While continuing systemic administration of liposomal amphotericin B, intratracheal instillation liposomal amphotericin B was performed by bronchoscopy three times a week. Although the respiratory conditions improved and the patient was discontinued on venovenous extracorporeal membrane oxygenation, he ultimately died of recurrence of malignant lymphoma.

**Conclusion:**

Intratracheal instillation of liposomal amphotericin B is safe, and liposomal amphotericin B instillation allowed a targeted high local drug concentration, which led to improvement in the invasive bronchial aspergillosis. In addition, since the patient was supported with venovenous extracorporeal membrane oxygenation, we were able to perform safe bronchoscopic debridement of airway lesions and intratracheal instillation of liposomal amphotericin B.

## Introduction

Invasive pulmonary *Aspergillus* (IPA) is a life-threatening opportunistic fungal infection that predominantly affects immunocompromised hosts [1]. Invasive bronchial aspergillosis (ITBA) is rare, accounting for 7% of IPA cases [2]. ITBA is an unusual form of IPA; in most cases, it is likely to be associated with IPA infiltration into the central airways [2–4]. In a case series and review, the mortality rate of ITBA is found to be high at about 40%, and 23.7% of ITBA patients require mechanical ventilator management [5]. However, to our knowledge, there are few reports of life-saving cases with venovenous extracorporeal membrane oxygenation (VV-ECMO) as rescue therapy in IPA and ITBA.

As per the proposal of the Infectious Disease Society of America (IDSA) guidelines in 2016 for *Aspergillus* infections, treatment with a mold-active triazole or intravenous liposomal amphotericin B (L-AmB) and bronchoscopic debridement of airway lesions in selected cases is recommended for ITBA, and especially for lung transplant recipients, adjunctive inhaled amphotericin (AmB) in the setting of ITBA is recommended [1], but as far as we have been able to determine, there were been no report of intratracheal instillation of L-AmB for ITBA.

Here, we report a case of ITBA and IPA that was successfully treated with VV-ECMO, and combined systemic and intratracheal instillation of L-AmB.

## Case presentation

**Fig. 1:**
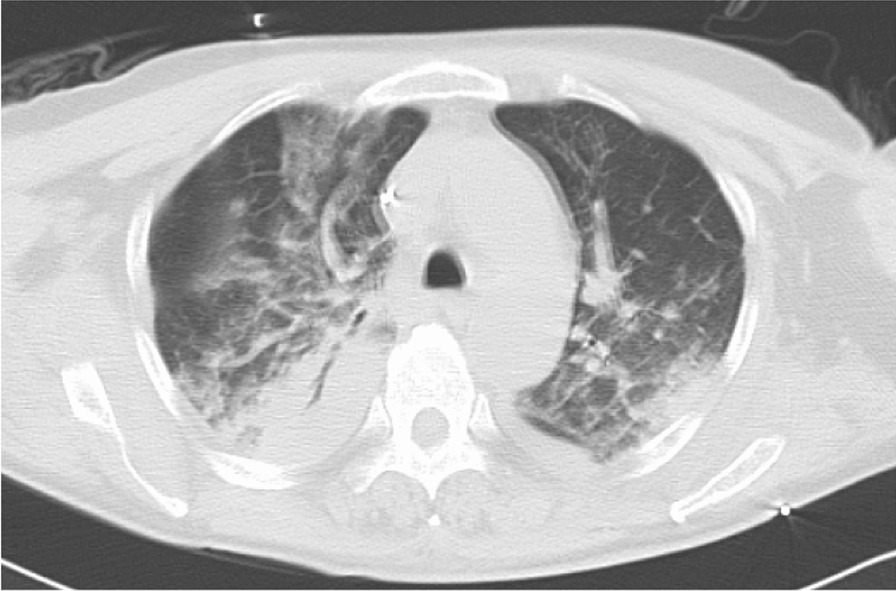
Chest computed tomography showed infiltration and bronchial thickening of right upper lobe

**Fig. 2:**
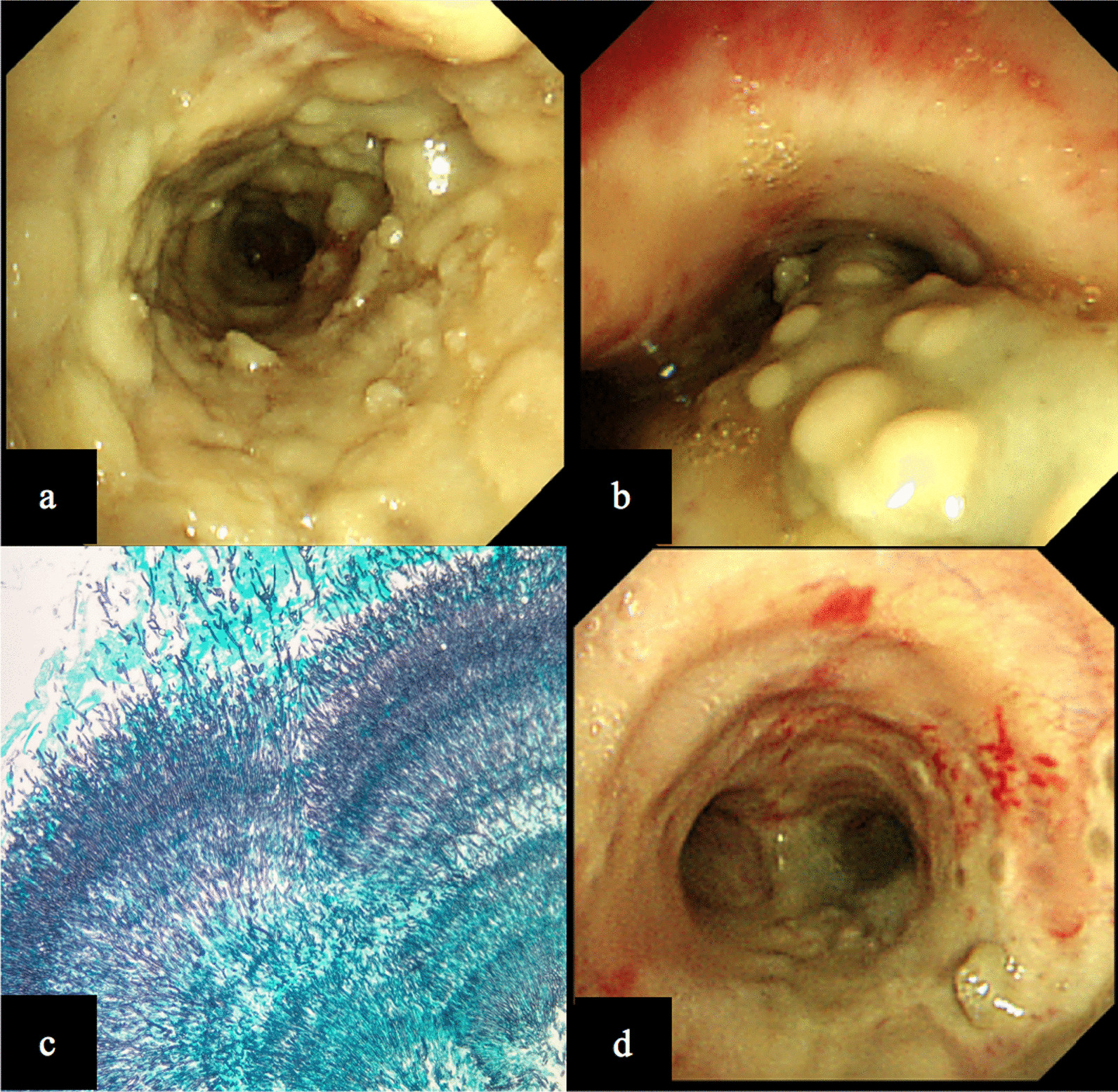
Bronchoscopy revealed diffuse wide raised pseudomembrane necrotizing tissue at right upper lobe (**a**). Pseudomembrane obstruction from the trachea to the left main bronchus resulted in atelectasis (**b**). Pathological finding of pseudomembrane showed numerous filamentous fungal hyphae (Grocott–Gomori’s methenamine silver stain, ×100) (**c**). After systemic and intratracheal instillation of liposomal amphotericin B, pseudomembrane necrotizing tissue disappeared (**d**)

A 61-year-old Japanese man who had no significant medical, family, or psycho–social history was admitted with a 2-week history of fever, whole-body lymph node swelling, and abdominal swelling caused by hepatosplenomegaly. He was diagnosed with peripheral T-cell lymphoma, not otherwise specified (PTCL-NOS), by histopathological analysis of the lymph nodes. On admission, his laboratory data showed pancytopenia, hyperbilirubinemia, acute kidney injury (AKI), and disseminated intravascular coagulation (DIC) (Table 1). Bone marrow aspiration revealed proliferating hemophagocytosis and lymphocytes, and we diagnosed the patient with lymphoma associated with hemophagocytic lymphoma (LAHS). The day after he was admitted to our hospital, he was treated with etoposide, prednisone, vincristine, cyclophosphamide, doxorubicin (EPOCH) chemotherapy. He responded to the chemotherapy; on day 9 after admission, DIC, hyperbilirubinemia, AKI, and hepatosplenomegaly improved. However, the period of bone marrow suppression from day 9 to day 19, lasted for 11 days, and he developed febrile neutropenia (FN). From day 9 after admission, he was treated for FN with meropenem 6 g/day and micafungin 100 mg/day, and was administered granulocyte colony-stimulating factor (G-CSF). His blood cultures were negative. On day 10, computed tomography of the chest revealed bilateral diffuse infiltration shadow of the lungs, and due to progressive respiratory failure, he required noninvasive positive pressure ventilation (Fig. 1). However, on day 15, type 2 respiratory failure progressed. The findings from a blood gas test were as follows; pH 7.18, PaO_2_ 77.6 mmHg, PaCO_2_ 72.3 mmHg, ${\mathrm{HCO}}_{3}^{ - }$ 26.8 mmol/L, and PaO_2_ to FiO_2_ (P/F) ratio 110. He underwent tracheal intubation for mechanical ventilation. Bronchoscopic findings revealed a widespread pseudomembranous necrotizing tissue formation covering the trachea and the bronchi, and numerous filamentous fungal hyphae infiltration were revealed in the pathologic examination (Fig. 2a–c). In addition, the β-d-glucan level was significantly increased to 1160 pg/mL, and the *Aspergillus* galactomannan antigen level [on enzyme-linked immunosorbent assay(ELISA)] was > 5.0. *Aspergillus fumigatus* was isolated from a pseudomembrane culture. We started L-AmB at 3 mg/kg/day. After intubation, he developed AKI with worsening of his general condition; therefore, we started continuous renal replacement therapy (CRRT). We performed daily bronchoscopy for pseudomembrane and necrotic tissue removal to release atelectasis. However, he was placed under deep sedation and fully controlled ventilation, and type 2 respiratory failure was worsened for atelectasis and obstructive pneumoniae. The findings from a blood gas test were as follows; pH 7.141, PaO_2_ 67.9 mmHg, PaCO_2_ 67.9 mmHg, ${\mathrm{HCO}}_{3}^{ - }$ 22.9 mmol/L, (FiO_2_ 0.8, PEEP 15 mmHg), and P/F ratio, 86. The patient showed severe hypoxemia (P/F = 86) under PEEP 15 mmHg, and severe respiratory acidosis with a Murray score of 4, so we initiated VV-ECMO as a reversible condition. Since severe respiratory failure occurred due to *Aspergillus* pseudomembrane that fills the tracheobronchial airway, systemic administration of only L-AmB was thought to be inadequate. After VV-ECMO, we continued to perform bronchoscopy three times a week for pseudomembrane removal and intratracheal instillation of aerosolized L-AmB. With the informed consent of his family, off-label use of L-AmB was administered via intratracheal instillation as a life saver. The L-AmB dose was referenced as an AmB attachment; 1 vial (50 mg) was dissolved in 10 mL of distilled water and 0.2–4 mL (1–20 mg) of the vial was added to it and further diluted (0.1–2 mg/mL of amphotericin B) in about 10 mL of distilled water. The L-AmB instillation allowed targeted high local drug concentration, which led to an improvement. In addition, we increased the systemic dose of the L-AmB to 6 mg/kg/day (the dose was increased to twice the normal dosage). Ten days after initiating VV-ECMO, a bleeding event from the gastric vestibular area was reported; however, VV-ECMO was successfully weaned after 14 days, with the improvement of lung function. Bronchoscopy revealed disappeared pseudomembrane necrotizing tissue (Fig. 2d). However, although ITBA improved, the patient developed an impairment of consciousness caused by central nervous system involvement of the lymphoma, at the same time, splenomegaly and enlarged inguinal lymph nodes were revealed again, soluble IL2 receptor was elevated (8527 U/mL), and uncontrollable metabolic acidosis had developed by lymphoma; the patient developed recurrent lymphoma and he died 50 days after admission.

**Table 1:** Laboratory data on admission

Hematology			Biochemistry			Arterial blood gas (room air)		
WBC	1800	/μL	T-Bil	7.6	U/L	pH	7.39	
Neut	94.0	%	D-Bil	5.5	mg/dL	PaCO_2_	30.3	mmHg
Lym	5.5	%	TP	5.3	mg/dL	PaO_2_	105.5	mmHg
Mono	0.5	%	Alb	3.1	mg/dL	${\mathrm{HCO}}_{3}^{ - }$	17.9	mmol/L
Eosino	0	%	AST	678	U/L	BE	− 6.2	mmol/L
Baso	0	%	ALT	312	U/L	Lactate	7.24	mmol/L
RBC	272 × 10^4^	/μL	LDH	3828	U/L			
Hb	9.0	g/dL	γGTP	155	U/L			
MCV	92.3	fl	ALP	297	U/L			
Plt	3.1 × 10^4^	/μL	BUN	47.4	mg/dL			
Coagulation			Cre	1.96	mg/dL			
PT–INR	1.23		eGFR	27.9				
APTT	67.4	s	UA	9.9	mg/dL			
Fib	86.0	mg/dL	Na	131	mEq/L			
FDP	27.3	mcg/mL	K	4.6	mEq/L			
Ddimer	11.9	mcg/mL	Cl	99	mEq/L			
			Ca	7.8	mg/dL			
			IP	5.4	mg/dL			
			GLU	85	mg/dL			
			CRP	5.33	mg/dL			
			HbA1c	5.7	%			
			sIL2R	26,824	U/mL			

*WBC* white blood cell, *Neu* neutrophil, *Lym* lymphocyte, *Mono* monocyte, *Eosino* eosinophil, *Baso* basophil, *RBC* red blood cell, *Hb* hemoglobin, *Plt* platelet, *PT* prothrombin time activity, *PT–INR* prothrombin time activity–international normalized ratio, *APTT* activated partial thromboplastin time, *Fib* fibrinogen, *FDP* fibrin/fibrinogen degradation products, *T-Bil* total bilirubin, *D-Bill* direct bilirubin, *TP* total protein, *Alb* albumin, *AST* aspartate aminotransferase, *ALT* alanine aminotransferase, *LDH* lactate dehydrogenase, *BUN* blood urea nitrogen, *Cre* creatinine, *eGFR* estimate glomerular filtration rate, *UA* uric acid, *Glu* glucose, *CRP* C-reactive protein, *HbA1c* hemoglobin A1c, *sIL2R* soluble IL2 receptor, *BE* base excess

## Discussion

Krenke et al. reported that ITBA in severely immunocompromised hosts with hematologic malignancies is characterized by a pseudomembranous overlying of the mucosal surface that is extensively involved in the lower airways. On the other hand, ITBA in those who have undergone a heart or lung transplant, is characterized by ulcerative types or plaque-like lesions in the bronchial walls [2]. In this case, the pseudomembrane necrotizing tissue caused severe respiratory distress that required ECMO support. Mario et al. reported that the overall in-hospital mortality rate of ITBA was 39.1%, with neutropenia [odds ratio (OR) 20.47; *p* < 0.001) and acute respiratory distress at presentation (OR 9.54; *p* = 0.002) being independent prognostic factors, indicating that the prognosis of ITBA in severely immunocompromised hosts with hematologic malignancies is worse than that of ITBA with other diseases [5]. In this case, severe neutropenia lasted for more than a week during bone marrow suppression following chemotherapy due to LAHS, and the patient had acute respiratory distress, and presented two poor prognostic factors. The IDSA guidelines recommend ITBA treatment with a mold-active triazole or intravenous L-AmB and bronchoscopic debridement of airway lesions in selected cases [1]. In this case, removal of atelectasis and obstructive pneumonia caused by pseudomembranes was considered necessary. However, bronchoscopic debridement of the the pseudomembrane may not be practicable due to high risk of hypoxia and progressive respiratory acidosis in cases of severe respiratory failure, even under mechanical ventilator control. Since the patient had severe respiratory failure, we started VV-ECMO management as rescue therapy, and then bronchoscopy could be performed safely and we were able to release atelectasis due to the pseudomembrane.

There is usually a delay in culture and pathology reports, and there is often a delay in fungal identification because some tests for species identification cross-react with other fungi. Also, in the present case, it took several days from the progression of type 2 respiratory failure to the diagnosis of fungal infection. Thair et al. reported that the cell-free DNA, which is collected for plasma next-generation sequencing (NGS) testing at the time of initial blood culture, identified a broad range of pathogens such as viruses, bacteria, and eukaryotic pathogens, which can provide valuable information to help clinicians better target antimicrobial therapy for septic patients [6]. It is hoped that NGS will become more accessible in clinical practice.

VV-ECMO is an effective treatment to support patients with severe respiratory failure who are unresponsive to conventional therapies [7]. *Aspergillus* spp. are identified more frequently (approximately 7%) in ECMO patients compared with patients without ECMO who are critically ill [8, 9]. However, to our knowledge, there is only one a single report of life-saving cases with VV-ECMO as rescue therapy in IPA [10], and there are no reports of cases with ECMO support in ITBA. In patients receiving VV-ECMO, drug pharmacokinetics may be significantly altered. Yanjun et al. reported a twofold increase in the standard total daily dose of both drugs to overcome low serum concentrations thought to be secondary to drug loss from ECMO circuit sequestration [11]. We upped the amount of L-AmB to 6 mg/kg/day, while the patient was treated with VV-ECMO and CRRT.

L-AmB is a unique lipid formulation of AmB. AmB binds to ergosterol in the fungal cell membrane, which leads to the formation of pores, ion leakage, and ultimately fungal cell death [12]. L-AmB is currently the most common type of AmB in distribution because intravenous AmB (Fungizone) is often complicated by nephrotoxicity [13]. The efficacy and safety of intratracheal inhaled L-AmB or AmB lipid complex for prophylaxis IPA have also been reported in hematopoietic stem cell transplant patients and in solid organ transplantation recipients [14–17], and efficacy and safety of intratracheal instillation of L-AmB was reported in treatment of endobronchial mucormycosis [18]. In addition, inhaling L-AmB for *Aspergillus* infection does not cause changes in the lipid content of the pulmonary surfactant [19]. Although only AmB has been approved for intratracheal inhalation and instillation, L-AmB is not indicated. This study was done in accordance with the institutional guidelines and the principles of the Declaration of Helsinki, which were ethically reviewed and approved by our hospital; informed consent was also obtained. This is the first report of an intratracheal instillation of L-AmB for ITBA. In this case, intratracheal instillation of aerosolized L-AmB by bronchoscopy was effective and safe for severe ITBA. At a time when AmB is not widely distributed, L-AmB is becoming more mainstream; therefore, intratracheal inhalation and instillation of LAmB will be even more necessary in the future to treat severe ITBA.

## Conclusions

In this case, we successfully treated severe respiratory failure associated with ITBA with timely implementation of systemic and intratracheal instillation of aerosolized L-AmB and maximal supportive care, including VV-ECMO. The L-AmB instillation allowed a targeted high local drug concentration, which led to an improvement. Because an intratracheal instillation of L-AmB is safe and effective, it could be a treatment option for ITBA. In addition, since the patient was supported with VV-ECMO, we were able to safely perform bronchoscopic debridement of airway lesions and intratracheal instillation of L-AmB.

## Data Availability

All data generated or analyzed during this study are included in this published article.

## Abbreviations

IPA: Invasive pulmonary *Aspergillus*
ITBA: Invasive bronchial aspergillosis
VV-ECMO: Venovenous extracorporeal membrane oxygenation
IDSA: Infectious Disease Society of America
L-AmB: Liposomal amphotericin B
PTCL-NOS: Peripheral T-cell lymphoma, not otherwise specified
AKI: Acute kidney injury
DIC: Disseminated intravascular coagulation
FN: Febrile neutropenia
LAHS: Lymphoma associated with hemophagocytic lymphoma
P/F: PaO_2_ to FiO_2_
CRRT: Continuous renal replacement therapy
NGS: Next-generation sequencing
